# Bioactive Surface Modifications on Bioresorbable Bone Screws: A Step Forward in Orthopedic Surgery

**DOI:** 10.3390/polym18010052

**Published:** 2025-12-24

**Authors:** Ainur G. Matveyeva, Olga P. Boychenko, Alexander P. Moskalets, Sergey S. Zakakuev, Nikolay A. Barinov, Alexandra S. Bogdanova, Olga V. Morozova, Dmitry V. Klinov, Dimitri A. Ivanov

**Affiliations:** 1 Lopukhin Federal Research and Clinical Center of Physical-Chemical Medicine of Federal Medical Biological Agency, 119435 Moscow, Russia; 2 Department of Chemistry, Lomonosov Moscow State University (MSU), 119991 Moscow, Russia; 3 Scientific Center for Genetics and Life Sciences, Sirius University of Science and Technology, 354340 Sochi, Russia; 4 Institut de Sciences des Matériaux de Mulhouse (CNRS UMR 7361), Université de Haute Alsace, 68100 Mulhouse, France

**Keywords:** implantable screws, polylactide, collagen, hydroxyapatite, bioactive coating, silver nanowires

## Abstract

Despite metals currently being widely used in orthopedic surgery, their mechanical properties significantly differ from the surrounding tissues and organs, causing low biocompatibility. Biodegradable, non-toxic, and non-immunogenic materials seem to be more convenient for clinical implementation. Our research was aimed at the construction of a polylactide screw covered with collagen, nanohydroxyapatite, and polylactide, with a variant including silver nanowires for antibacterial properties, as well as the analysis of their physico-chemical and biological properties. Adherent human osteosarcoma cells (2T line) were shown to grow on the porous surface layers. A cytotoxicity assay using WST1 revealed the non-toxic nature of the coatings and showed an increase in cell adhesion and proliferation. Safety and efficacy were also evaluated in vivo with the coated screws implanted into the metatarsal bones of minipigs. Histological analysis at 29 and 58 days post-screw-implantation revealed that the coated samples accelerated bone tissue regeneration compared to uncoated controls. This was evidenced by a higher bone-to-granulation tissue ratio, reduced inflammatory cell counts, and increased osteoblast/osteoclast activity at the early stage during the initial days after implantation. The results confirm that the developed bioactive coatings enhance biocompatibility and osteointegration.

## 1. Introduction

The internal fixation of damaged bones and tendons significantly affects their proper fusion and regeneration. Bare metals are widely used for screws in traumatology and orthopedics. Despite their widespread use in traumatology and orthopedics, metal implants have several disadvantages. Most important is the difference in mechanical properties of the metals and the surrounding tissues and organs, thus causing inevitable removal associated with a risk of repeated fractures and other undesirable consequences [1].

Moreover, metals typically have higher tensile stiffness than fused bones. For example, the Young’s modulus of trabecular bone is about 60 GPa, while the Young’s modulus of cobalt–chrome alloys ranges between 100 and 200 GPa [2]. Such a discrepancy in stiffness may hinder proper bone regeneration due to uneven strain distribution [3,4], leading to an increased probability of repeated fractures [5,6].

To overcome the aforementioned limitations of bare metal implants, bioresorbable implants based on synthetic polymers, such as polyglycolide, polylactide, polydioxanone, their copolymers, and others have been used since the 1980s. These polymers had been employed in clinical practice previously, but in the form of resorbable surgical sutures. They demonstrated high biocompatibility and low toxicity. Bioresorbable polymeric screw materials promoted fracture healing by stimulating periosteal callus formation and allowing for gradual strain transfer [7,8]. However, early research in this area has revealed significant problems and complications [2], including premature destruction of the implant material, which could lead to repeated fractures, and the occurrence of sterile fistulas around or near the implantation area [9,10].

More than forty years of research and clinical observations have expanded the data on the benefits and drawbacks of various biodegradable materials and constructions. Particularly, poly(L-lactide) degrades slowly, but this feature has a dual effect. On the one hand, long-term degradation results in gradual strain transfer, which favors poly(L-lactide) over metals [11]. On the other hand, an increase in acidic degradation products may lead to osteolysis [12]. Despite the stability and functionality of polylactide screws, they cannot always be used as a substitute for metal ones, e.g., anterior cruciate ligament reconstruction. Long-term application of such screws may cause large bone tunnel formation [13,14].

An accumulation of acidic decomposition products, notably lactic and glycolic acids, might affect the osteoblasts’ activity, lowering their proliferation, accelerating differentiation, and hampering the regeneration process [15]. Adding minerals, such as hydroxyapatite or β-tricalcium phosphate, to polymers refines the material’s osteoconductive features [16]. Calcium cations released by these minerals reduce local acidification, soothe inflammation, and promote bone growth and integration [17,18,19]. Composite screws, containing polylactide-co-glycolide (or poly-L-lactide) and tricalcium phosphate (or hydroxyapatite), effectively participate in bone recovery, while preventing bone tunnels’ extension and cyst formation [20,21,22,23].

Modifying the physico-chemical and biological parameters of bioresorbable composite screws by using synthetic copolymers and bioceramics still lacks safety and efficiency. Researchers continually face tunnel expansion, irreversible tissue damage, osteolysis, foreign body reactions, synovitis, effusion, implantation-associated bacterial infections, and premature biomaterial resorption [24].

Polylactide electrospun nanofibrous materials coated with bioceramic hydroxyapatite, bioactive glass, or tricalcium phosphate particles have shown great osteoconductive and osteointegrative potential in rat bone tissue regeneration [25,26]. This previous study emphasizes the role of implant surface morphology in effective bone regeneration.

In the present study, we developed a method of fabrication for polylactide screws and their surface modification. Polylactide has a wide range of applications in medicine due to its excellent biocompatibility and biodegradability. This material is implemented for the production of pharmaceuticals and their coatings, encapsulation technologies, and the manufacture of a variety of medical devices, including fixation rods, plates, pins, screws, and sutures. Polylactide is also used in the manufacture of bioresorbable medical implants, tissue regeneration components, drug delivery systems, and membrane coatings [27]. We designed the surface structuring of the screws so that bone tissue would grow through the implant. The in vitro biocompatibility of the bioactive surface was observed in human osteosarcoma 2T cells. The bioactive coating of the screws also accelerated bone tissue regeneration in the early stages of healing compared to the uncoated screws.

## 2. Materials and Methods

### 2.1. Scanning Electron Microscopy (SEM)

SEM experiments were performed on a Merlin electron microscope with a GEMINI II Electron Optics column (Zeiss, Oberkochen, Germany). The samples were generally coated with a thin layer of Au/Pd. Images were obtained at a 1–5 kV accelerating voltage and a current of 100–300 pA. The obtained images were analyzed using FIJI software (ver. 1.54p).

### 2.2. Atomic-Force Microscopy (AFM)

AFM experiments were carried out using ultra-sharp probes on an Ntegra Prima (NT-MDT, Moscow, Russia) microscope in the tapping mode (attractive regime). The typical scanning frequency was 1 Hz, and the pixel resolution was about 1 pixel/nm. Nova Image Analysis software (ver. 1.1.0) was used for standard image processing and the presentation of AFM images. SPM Image Magic (ver. 1.1.2.2782) was employed for analyzing object heights.

### 2.3. Screw and Disk Fabrication

The screws were made of Ingeo 4032D (NatureWorks, Plymouth, MN, USA) polylactide and molded using a laboratory twin-screw extruder, Haake Minilab II (Thermo Scientific, Vienna, Austria), at a temperature of 205 °C and a screw rotation rate of 60 rpm. These parameters were chosen to minimize the formation of air cavities, prevent the degradation of the polymer, and facilitate rapid solidification in the mold. The molded screws had dimensions of 7.8 × 23 mm. In a similar way, disks with a thickness of 1 mm and a diameter of 5.5 mm were prepared from Ingeo 4032D polylactide.

### 2.4. Nanohydroxyapatite (nHA) Formation

A method for obtaining hydroxyapatite in the form of nanocrystals (nHA) was performed according to a modified procedure from ref. [28]. Briefly, 16.5 mL of a 0.75 M solution of calcium L-lactate (Sigma Aldrich, Saint Louis, MO, USA) was poured into 25 mL of a 0.25 M (NH_4_)_2_HPO_4_ (Chimmed, Moscow, Russia) solution in water at room temperature under vigorous stirring, and then, after setting the pH = 10 with concentrated ammonia, was left to age for 2.5 h. The obtained suspension was centrifuged for 30 min at 2300× *g*, the precipitate was diluted with 30 mL of isopropyl alcohol (Chimmed, Moscow, Russia), and properly mixed. This washing procedure with isopropanol was repeated three times. Finally, the concentrated suspension was vacuum-dried at 56 °C.

According to the SEM data, the obtained hydroxyapatite nanoparticles (nHA) had a size of approximately 30 × 10 nm. After drying, they partially aggregated, forming larger particles. Their subsequent ultrasonic dispersion resulted in the appearance of a larger fraction, with a size of up to 200 nm (Figure S1).

### 2.5. Silver Nanowires Synthesis

Briefly, 5 g of polyvinylpyrrolidone with a molecular weight of 360 kDa (Sigma Aldrich, Saint Louis, MO, USA) was dissolved in 160 mL of glycerol (Chimmed, Moscow, Russia) with continuous stirring. Subsequently, 1.32 g of AgNO_3_ (Chimmed, Moscow, Russia) was added and stirred until fully dissolved. This was followed by the addition of 8.75 mL of a NaCl (Sigma LifeScience, Saint Louis, MO, USA) solution in glycerol 5.57 mg/mL. The mixture was then heated to 145 °C for 45 min, and finally to 170 °C for 15 min.

After cooling, the solution was diluted by half with water and then centrifuged for 15 min at 10,000× *g*. The precipitated silver nanowires (AgNW) were diluted with 160 mL of distilled water, thoroughly mixed, and underwent the washing process with water twice. According to SEM, AgNW had a thickness of around 30–50 nm, and a length of 2–20 µm; a minor impurity of nanoparticles of a different morphology (Figure S2) was also present.

### 2.6. Bioactive Coating Application

The bioactive coating of the 5.5 mm-diameter disks was implemented using three different methods: BAC1, BAC2, and BAC3. In the BAC1 method, the disks were dipped into a suspension of polylactide Purasorb PL32 (Corbion, Amsterdam, The Netherlands), collagen (BioUltra, Sigma Aldrich), and prepared nHA in HFIP, dried after removing the excess suspension, and the process was repeated once more. Conversely, the BAC2 method involved an additional step of immersing the disks in isopropanol at 80 °C for 10 min before drying. Finally, the BAC3 method comprised applying a 0.5 mm layer of the suspension, immersing the disks in isopropyl alcohol at 80 °C for 10 min, followed by drying. All samples were then finally dried in a vacuum at 54 °C to a constant weight. The samples made by these methods are further referred to as Col/nHA/PLA_1, Col/nHA/PLA_2, and Col/nHA/PLA_3. The bioactive-coated disks obtained from these methods were then placed in a 96-well plate for testing in MTT.

### 2.7. Cytotoxicity Investigation In Vitro

Toxicity was assessed using a culture of human osteosarcoma 2T cells obtained from the Collection of Tissue Cultures at the National Research Center for Epidemiology and Microbiology, named after Honorary Academician N.F. Gamaleya, under the Ministry of Health of Russia in Moscow. The 2T culture was polymorphic, comprising epithelial-like cells with nearly circular nuclei of varying sizes.

The 2T cell line, which mimics bone tissue growth, was cultured at 37 °C and 5% CO_2_ for 1, 3, 7, and 10 days on 5.5 mm disks to investigate the cytotoxic properties of the developed biocoatings. The culture medium contained 8% fetal bovine serum (FBS HyClone) (Thermo Scientific, Waltham, MA, USA), 100 units/mL penicillin, and 100 units/mL streptomycin, and was refreshed every third day. Cells were seeded at a density of approximately 3000 cells per well of a 96-well plate in 300 μL of culture medium.

Quantitative assessment of in vitro cytotoxicity was performed using the MTT assay. A sterile WST-1 solution (BioVision, Exton, PA, USA) was added in a 1:10 ratio to the medium in each well, and the samples were then incubated for 2 h. The optical density, measured at λ = 450 nm, served as an indicator of the number of viable cells.

### 2.8. Sterilization

After the application of the bioactive coatings, the prepared screws were packaged in a 70 × 125 mm bag made of polyethylene terephthalate/polypropylene film from Danaflex-NANO LLC (Moscow, Russia), sealed with crepe soft paper (Sterisheet packaging material for medical air, steam, gas, plasma, and radiation sterilization) from Arjowiggins SAS (Palalda, France). Subsequently, the manufactured batch of screws underwent radiation sterilization in compliance with the national standard [29] (irradiation dose of 15 kGy).

### 2.9. In Vivo Safety and Resorptive Properties of the Coated Screws

Experiments were carried out on male minipigs free from: *Actinobacillus pleurovneumoniae*, *Streptococcus b-haemolyticus*, *Streptococcus suis*, *Pasteurella multocida*, *Staphylococcus hyicus*, *Hemophilus parasuis*, *Mycoplasma hyopneumonia*, *Salmonella* spp., *Corynebacterium* spp., *Yersinia enterocolitica*, *Leptospira* spp., and endo- and ectoparasites (ticks, helminths, and protozoa). The animals were divided into three groups of 2 males each: group No. 1—control (denoted further in the text as M); Groups No. 2 and No. 3—tested objects T1 and T2, respectively (Table 1). The body weight of the animals was 38.4 ± 1.5 kg. Before the experiment, the animals were kept in enclosures. The clinically healthy animals were kept for 4 days to adapt to the experimental conditions. During this period, the animals were monitored for clinical status by visual inspection every day. The distribution of animals into groups was based on data on the body weight of the animals.

Animals were kept in accordance with Directive 2010/63/EU of the European Parliament and of the Council of 22 September 2010 on the protection of animals used for scientific purposes [30] and Guidelines for the Care and Use of Laboratory Animals [31].

Implantation of the screws was carried out in pigs previously anesthetized with a mixture of Zoletil 100 and Rometar at a dose of 5 mg/kg and 2 mg/kg administered intramuscularly.

The studied screws with or without coating were implanted into the animals’ 2nd or 3rd metatarsal bone of the right pelvic limb. In the area of the metatarsal bones of the right pelvic limb, hair was removed, and the skin was treated with a skin antiseptic. Using a scalpel, a U-shaped incision was made, and the superficial tissues were separated to expose the surface of the metatarsal bone (Figure S3). Next, the sites for the two screws were prepared ~7 mm from the distal edge of the femur using a low-speed drill with a 7.4 mm diameter round bur under continuous irrigation with sterile saline (0.9%). Then the test screws were placed into the holes, after which the wound was sutured layer by layer and treated with antiseptics. After surgery, the animals received an antibiotic (Amoxicillin 15–15 mg/kg) and an analgesic (Tramadol—5 mg/kg) intramuscularly for 3 days. The characteristics of the experimental groups and the experimental design are presented in Table 1.

The animals were deprived of food 6–12 h before anesthesia on the days of formation of the wound surface and before collecting material for histological examination. Body weight was recorded on the first day before the formation of wounds and then once every two weeks (to assess the general condition of the animals). The pigs’ weighing was carried out on electronic platform scales VPA-100-1 (VIK Tenzo-M, Moscow, Russia). The smallest weighing limit was 0.4 kg, along with the following parameters: maximum weighing limit—100 kg; verification division step—0.01 kg; accuracy class—III.

Clinical observation of the animals was carried out daily for 57 days. Clinical examination of animals was carried out once every two weeks. The behavior of the animals in the holding box (while the animal was awake), the condition of the skin, the presence of discharge, and other changes in the wound surface (sites of implantation of the studied objects) were assessed.

On the 29th and 58th days of the experiment, one animal from each group was euthanized, and part of the metatarsal bone with implanted screws was removed for subsequent pathomorphological and histological studies.

In accordance with Directive 2010/63/EU of the European Parliament and of the Council for the Protection of Animals Used for Scientific Purposes, of 22 September 2010, the animals were euthanized by stunning by passing an electric current through the animal, followed by cutting the main great vessels. This type of animal euthanasia involved a minimum of pain, suffering, and distress and was carried out by competent staff.

### 2.10. Pathomorphology

There were no animals that died during the experiment; no unscheduled necropsy procedures were performed.

On the 29th and 58th days of the experiment, during the planned euthanasia of experimental animals, a necropsy procedure was carried out for each animal with the completion of a macroscopic examination protocol. Necropsy was performed under the direct supervision of a pathologist. Immediately after the planned euthanasia and necropsy, a part of the metatarsal bone with implanted screws was removed for subsequent pathomorphological examination (examination of the implantation site) and transfer for histological examination.

The sample was fixed for 24 h in a 10% neutral formaldehyde solution before being embedded in paraffin in accordance with the generally accepted protocol for histological examination [32]. Subsequently, sections of 5–7 μm in thickness were produced and stained with hematoxylin and eosin [33]. The histological preparations were examined using an Accu-Scope 3000 SERIES light optical microscope (Accu-Scope, Commack, NY, USA) at magnifications of 40, 100, 200, and 1000. A Toupcam UCMOS05100KPA (ToupTek, Hangzhou, China) digital camera and ToupView 3.7.7892 (ToupTek, Hangzhou, China) software were used for microphotography.

Due to the removal of implants, it was extremely difficult to accurately determine the boundary of their contact with newly formed tissues.

The study evaluated the process of bone tissue regeneration as well as the surrounding tissue’s response to a foreign body (implant) in terms of the intensity of the inflammatory response [34,35,36]. Quantification was performed on osteoblasts and osteoclasts at the location of newly formed bone as well as inflammatory cells (lymphocytes, leukocytes, and macrophages) in the nearby granulation tissue. At a magnification of ×400, counting was performed in ten fields of vision in the region with the most noticeable alterations. Furthermore, using images captured at ×40 magnification, the ratio of granulation tissue to bone tissue was evaluated using the VideoTesmer software (ver. 5.2).

Descriptive statistics were used to analyze the data due to the small sample at all key points.

### 2.11. Statistical Analysis

All statistical results are presented as mean ± standard deviation, unless otherwise indicated.

## 3. Results

### 3.1. Formation of Bioactive Coatings

**Figure 1:**
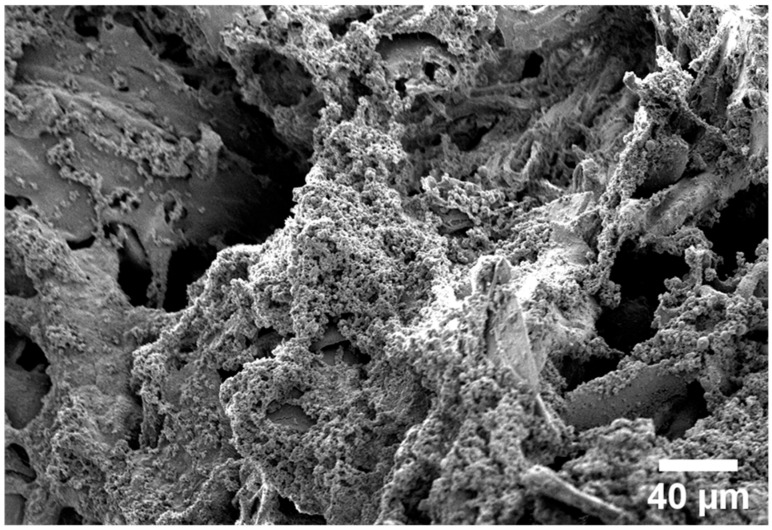
SEM image of the bioactive coating obtained by precipitating the Col/nHA/PLA (1:2:1 by mass in HFIP) suspension in isopropanol.

Two methods were used to form the nanostructured bioactive coating on the surface. The first involved applying nanofibers obtained by electroforming, and the second was the self-assembly of nanostructures on the surface of the screws. For applying the nanofibers, the following procedure was used with the electrospinning method. The obtained nHA was mixed with PLA and collagen in HFIP solution to create a homogeneous suspension, from which non-woven matrices were obtained using the electrospinning method to investigate the toxicity of the new biomaterials. The matrix composition (12.5% (by mass) collagen, 50% nHA, 12.5% PLA) was selected to fulfill two important functions of the developed bioactive coating: cell adhesion to the surface (due to the presence of collagen) and osteogenicity (due to the substantial content of calcium phosphates in the form of nanohydroxyapatite). Figure 1 shows that even large nHA aggregates were incorporated inside the polylactide and collagen structures.

**Figure 2:**
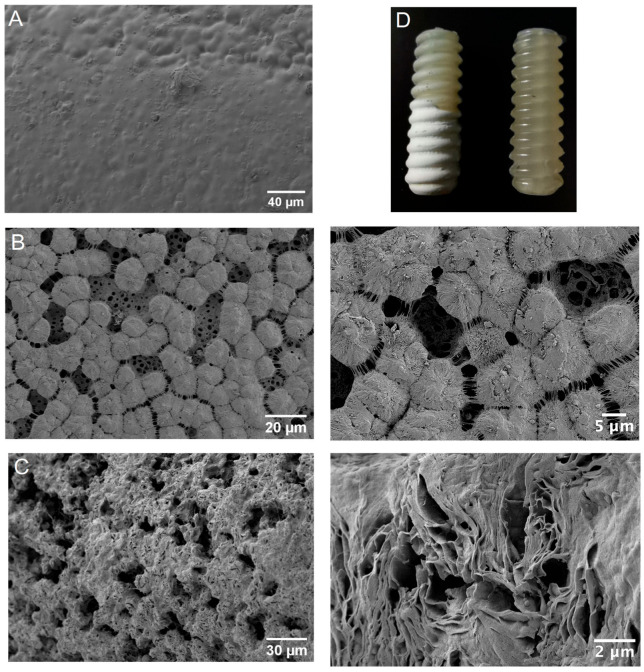
SEM image of the surface of a thin (approximately 10 µm) and thick (approximately 100 µm) bioactive coating ((**B**) and (**C**), respectively) compared with the surface of the original screw (**A**). Photographic images of the fastening screw with a thin (left, top) and thick (left, bottom) layer of nanostructured bioactive coating in comparison with the uncoated screw (right) are shown in (**D**), with explanations given in the text.

The deposition of a polylactide–collagen–hydroxyapatite mixture onto the screws led to the formation of a bioactive coating (Figure 2). Compared to the surface of the initial screw, the coating exhibited high porosity and contained a number of pores. The pore size varied widely, ranging from hundreds of nm to tens of µm. Notably, the porous structures resulting from phase separation in the system, upon the addition of isopropanol, had dimensions comparable to the size of a cell (2–10 µm), which allowed for the penetration deep into the bioactive coating and strong adhesion to it.

Since the coating was highly porous, its mechanical strength was expected to be low. We found that thick coatings indeed exhibited low mechanical strength under shear stress, leading to the delamination of the outer layer when the screw was implanted into the bone hole. However, after scratching the outer layer, a thin bioactive layer remained on the screw surface, which still promoted cell adhesion. This little disadvantage of the thick coating could potentially be eliminated, for example, by using hydroxyapatite nanofibers or nanorods that would act as a reinforcing material.

Additionally, to impart antibacterial properties to the surface, half of the screws with the bioactive coating were treated with a suspension of silver nanowires (Figure S4). For this purpose, silver nanowires were introduced into the solution for forming the bioactive coating [2,8].

### 3.2. Biocompatibility Studies of Bioactive Coatings In Vitro

To assess the biocompatibility of the developed bioactive coatings, they were formed on a flat surface for convenient observation under a fluorescent microscope.

**Figure 3:**
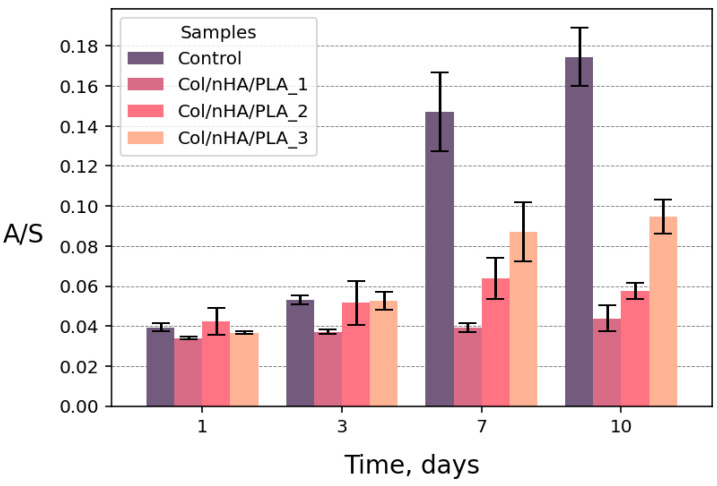
Evaluation of the enzymatic activity of living 2T cell culture obtained using MTT. The optical density value is normalized to the well/sample area.

Initially, a toxicity assessment was conducted for the adherent human osteosarcoma cell culture 2T. The 2T culture cells actively adhered to and proliferated on the surfaces of the developed bioactive coatings. For the quantitative assessment of material biocompatibility, the MTT assay was used to evaluate the enzymatic activity of all living cells. A comparison of the MTT assay response between controls and samples was performed for optical density values normalized to the well/sample area. Figure 3 demonstrates the sequential growth of cell activity in all samples, including the control. It is worth noting that the growth occurred at different rates. It can be assumed that on a less smooth surface than cell culture plastic polystyrene of tissue culture grade, cells adhered more slowly. Nevertheless, all samples showed no toxicity to the 2T cell line, with the Col/nHA/PLA_3 sample being the most biocompatible.

Thus, it can be stated that the nanostructured bioactive coatings for the surfaces of fastening devices developed in the present study were non-toxic and promoted the rapid growth and proliferation of the cultured cells under investigation.

### 3.3. In Vivo Biocompatibility Studies

Two types of polylactic acid screws with biocompatible coatings were selected as objects for in vivo studies—“collagen/nHA/polylactic acid” (Col/nHA/PLA, T1) and “collagen/nHA/polylactic acid/silver nanowire” (Col/nHA/PLA/AgNW, T2). Additionally, an uncoated screw (M) was used as a control.

The safety assessment of the selected screws was conducted on miniature pigs. Miniature pigs are the most clinically relevant model for studying the safety and resorptive properties of the investigated objects due to their anatomical and physiological similarity to humans [37,38]. While the bone composition is relatively conservative, it differs among species; canine and porcine bones are compositionally and densitometrically closer to human bones, whereas rats show differences in these parameters. Additionally, bone regeneration decreases, and morphology changes differently with age depending on the lifespan. This was particularly important for determining the size of the formed defect [39,40]. Mature miniature pigs over 12 months of age were used in this study [40].

The most sensitive stage of healing in terms of implant biocompatibility is the first month of recovery after surgery. A cascade of cellular events from blood clotting to the formation of immature woven bone is initiated on the implant surface that comes into contact with the biological environment. Within the first week, the differentiation of osteoblasts is stimulated by the production of osteogenic factors, growth factors, and cytokines. Primary bone, including trabecular tissue, is replaced by parallel-fibered and/or lamellar bone and bone marrow. Between 1 and 2 weeks, the bone tissue responsible for the primary mechanical stability of the implant is resorbed and replaced by newly formed bone laterally adjacent to the implant region. By 4 weeks, secondary stability is achieved with a significant number of osteocytes. Bone tissue regeneration around the implant area within the first two months after surgery was investigated in this study.

#### 3.3.1. General Condition of the Animals

Throughout the experiment, no deviations in the health status of the animals were observed, except for the first 3–5 days after the implantation of the test objects, when a decrease in appetite was noticed. Additionally, from the first 5–7 days, mild lameness in the right hind limb (at the time of placing weight on the operated limb, animals did not fully rely on it, keeping it elevated) and slight swelling were observed, which was normal for this type of operation.

A detailed examination of the surgical site was conducted every two weeks. A typical pattern of normal wound healing processes without purulent-inflammatory manifestations was observed. There were no signs of implant site infection throughout the experiment. A decrease in body weight was noted 2 weeks post-operation, associated with decreased appetite during the recovery period. Nevertheless, subsequent weight measurements showed a positive trend. Changes in body weight as an integral indicator of health were not observed.

#### 3.3.2. Pathomorphological Data

Histological examination of the material taken at the first study point (29th day of the experiment), directly at the site of implant placement, revealed processes of bone tissue regeneration. The defect was filled with fibrous-granulation tissue, consisting of loose connective tissue with numerous small capillaries, moderately expressed infiltration by fibroblasts and inflammatory cells, mainly lymphocytes and macrophages. From the intact trabecular bone, proliferating osteoid trabeculae (“bone beams”) with an indistinct layered pattern extended towards the defect. The intertrabecular space was filled with reticular-fibrous tissue where the proliferation of periosteal bone tissue cells—osteoblasts and osteoclasts—located on trabecular surfaces, was observed.

Two months after the operation, on the 58th day of the experiment, the area of newly formed bone tissue significantly increased in all groups, compared to the previous time point. Similarly to the 29th day, the groups with implanted test screws had more newly formed bone tissue than the control group with the control screw. Table 2 provides the ratio of bone tissue to granulation tissue. The higher this ratio, the more pronounced the regeneration process. On the 29th day, the groups with implanted test screws had more newly formed bone tissue than the control group.

Table 3 presents quantitative data on the determination of osteoblasts, osteoclasts in the site of new bone tissue formation, as well as inflammatory cells (lymphocytes, leukocytes, and macrophages) in the adjacent granulation tissue on the first and second stages of the experiment (10 fields of view from each screw were examined). On the 29th day of the experiment, a reduction in the number of inflammatory cells and an increase in the number of osteoblasts/osteoclasts involved in bone tissue regeneration processes [41,42,43] were observed in the groups with the test screws, compared to the control screw.

**Figure 4:**
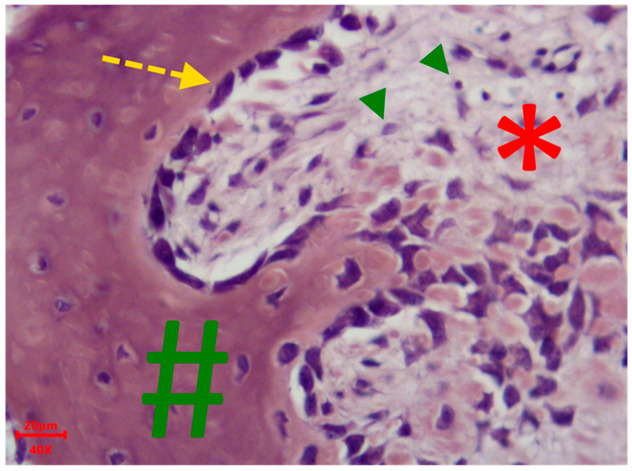
Cross-section of the new bone tissue formation area. Mini pig #3.1. Hematoxylin/eosin staining. *—granulation tissue, #—bone tissue, yellow arrows—osteoblasts, green arrows—inflammatory cells. Magnification ×400.

**Figure 5:**
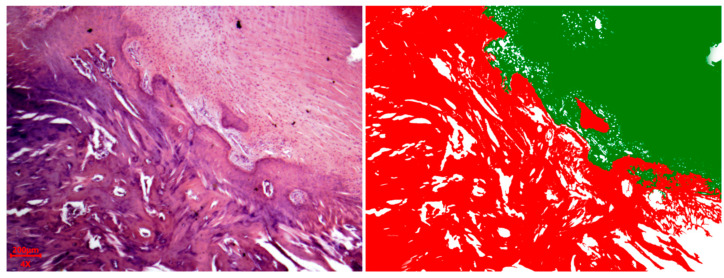
Cross-section of the area of new bone tissue formation on the 58th day of the experiment. Mini pig #2.2, screw #1. **Left**—hematoxylin/eosin staining. **Right**—processed photograph; green areas—young bone tissue, red areas—granulation tissue. Magnification ×40.

On the 58th day of the experiment, a well-defined trabecular structure was observed in the young bone tissue. The osteoid resembled mature bone, with areas showing circumferential lamellar-like structures akin to Haversian canals. The area of fibrous-reticular tissue decreased. The number of osteoblasts decreased, and single osteoclasts were visible in the field of view (see Figure 4). Figure 5 shows an example of a cross-section of the area of new bone tissue formation on the 58th day of the experiment.

On the 58th day of the experiment (Table 3), the group with the control screw showed a decrease in inflammation compared to the 29th day of the experiment and an increase in the number of osteoblasts. In the groups with the test screws, a decrease in both inflammatory cells and osteoblasts/osteoclasts was observed, indicating a gradual transition to the next stage of regeneration compared to the 29th day, in line with the literature [41,42,43].

## 4. Discussion

In the present study, prototypes of orthopedic screws made of polylactide with nanostructured bioactive coating were developed and investigated for biocompatibility. The thickness of the resulting coating can be widely varied (from ten to hundreds of micrometers), along with its porosity. The obtained coating demonstrated good adhesion to the surface of the orthopedic screw. The results showed the absence of a cytotoxic effect of the bioactive coatings on the culture of osteosarcoma 2T cells. Additionally, it demonstrated active cell growth after attachment to the developed coating.

The safety of the investigated fastening devices was also tested in vivo on miniature pigs. The absence of general toxic effects of the tested materials was demonstrated. In the first 5–7 days, the animals showed lameness in the right hind limb and swelling at the site of screw implantation, which was characteristic of this type of operation. A decrease in appetite was observed in the first week, resulting in a decrease in body weight on the 14th day of the experiment, followed by subsequent normalization. A characteristic pattern of normal wound healing was observed without any purulent or inflammatory manifestations. Throughout the experiment, the overall condition of the animals remained normal. Pathomorphological examination using the tested screws showed accelerated regeneration processes compared to the control polylactide screw, reflected in the increased ratio of bone tissue to granulation tissue on the 29th and 58th day of the experiment, a decrease in inflammatory cells, and a simultaneous increase in the number of osteoblasts/osteoclasts on the 29th day of the experiment.

Based on the study results, it can be concluded that polylactide screws for implantation with bio-compatible coatings (collagen/nanohydroxyapatite/polylactide and collagen/nanohydroxyapatite/polylactide/silver) do not have a general toxic effect on the animals’ bodies. Furthermore, the use of screws with bioactive coatings led to accelerated regeneration processes compared to control screws without coatings.

## Figures and Tables

**Table 1:** Parameters of the experimental groups.

№	Number of Animals in the Group	The Research Object [Object Code in the Study]	Implantation Site	Euthanasia
1	2	Control—polylactide screw without coating [M]	2 screws each in the 2nd or 3rd metatarsal bone of the right pelvic limb	After 1 month, one animal from each group; after 2 months, the one remaining
2	2	Object № 1—screw with coating “BAC2” [T1]	2 screws each in the 2nd or 3rd metatarsal bone of the right pelvic limb	
3	2	Object № 2—screw with coating “BAC2/AgNW” [T2]	2 screws each in the 2nd or 3rd metatarsal bone of the right pelvic limb	

**Table 2:** Ratio of bone tissue to granulation tissue.

Group	Object Code	Screw Number	Regeneration Indicators	
			Inflammatory Cells	
			Each Screw (*n* * = 3)	Total (*n* * = 6)
1st stage				
1	M-2/44/22	1	1.02 ± 0.096	0.97 ± 0.086
		2	0.93 ± 0.050	
2	T1-2/44/22	1	1.19 ± 0.059	1.16 ± 0.080
		2	1.13 ± 0.096	
3	T2-2/44/22	1	1.26 ± 0.122	1.22 ± 0.097
		2	1.18 ± 0.058	
2nd stage				
1	M-2/44/22	1	1.26 ± 0056	1.17 ± 0.111
		2	1.08 ± 0.058	
2	T1-2/44/22	1	1.35 ± 0.119	1.41 ± 0.129
		2	1.46 ± 0.137	
3	T2-2/44/22	1	1.41 ± 0.159	1.46 ± 0.132
		2	1.50 ± 0.108	

*—number of fields of view.

**Table 3:** Quantitative data on the determination of osteoblasts and osteoclasts at the site of new bone tissue formation, including inflammatory cells.

Group	Object Code	Screw	Regeneration Indicators					
			Inflammatory Cells		Osteoblasts		Osteoclasts	
			Each Screw *n* * = 10	Total *n* * = 20	Each Screw *n* * = 10	Total *n* * = 20	Each Screw *n* * = 10	total *n* * = 20
1st stage								
1	M-2/44/22	1	27.8 ± 8.75	31.0 ± 8.74	16.6 ± 4.58	14.5 ± 4.94	0.5 ± 0.71	1.2 ± 1.01
		2	34.1 ± 7.91		123 ± 4.50		1.9 ± 0.74	
2	T1-2/44/22	1	25.6 ± 8.45	25.0 ± 7.95	20.2 ± 6.80	18.7 ± 6.95	0.9 ± 0.74	1.3 ± 0.79
		2	24.4 ± 7.83		17.2 ± 7.13		1.6 ± 0.70	
3	T2-2/44/22	1	24.1 ± 11.99	26.2 ± 10.74	21.5 ± 4.97	19.8 ± 5.3	1.6 ± 1.17	1.7 ± 0.98
		2	28.3 ± 9.49		18.1 ± 5.30		1.8 ± 0.79	
2nd stage								
1	M-2/44/22	1	24 ± 6.57	19.5 ± 9.5	21.3 ± 5.40	20.9 ± 5.54	1.2 ± 1.03	1.0 ± 0.94
		2	14.9 ± 10.07		20.5 ± 5.95		0.7 ± 0.82	
2	T1-2/44/22	1	19.4 ± 5.34	18.9 ± 7.39	17.2 ± 5.05	15.3 ± 6.00	0.4 ± 0.52	0.4 ± 0.50
		2	18.3 ± 9.29		13.3 ± 6.48		0.4 ± 0.52	
3	T2-2/44/22	1	18.4 ± 7.89	17.4 ± 6.92	16.4 ± 4.95	17.7 ± 4.58	0.5 ± 0.71	0.6 ± 0.76
		2	16.4 ± 6.04		18.9 ± 4.04		0.6 ± 0.84	

*—number of fields of view.

## Data Availability

The original contributions presented in this study are included in the article/Supplementary Materials. Further inquiries can be directed to the corresponding authors.

## Supplementary Materials

The following supporting information can be downloaded at: https://www.mdpi.com/article/10.3390/polym18010052/s1, Figure S1: SEM image of hydroxyapatite nanoparticles and AFM image of hydroxyapatite nanoparticles after the drying and redispersion procedure; Figure S2: SEM image of silver nanowires; Figure S3: (A) U-shaped incision, exposed surface of the metatarsal bone; (B) Location of the implanted screws in the metatarsal bone; (C) Closing the wound, suturing; (D) Treatment of the seam and tissues adjacent to the seam with “Aluminum-Spray”; Figure S4: SEM image of the surface of thick (top) and thin (bottom) bioactive coatings with deposited silver nanowires. The positions of some nanowires are indicated by arrows.
