# Supplementary material for: Bioactive Surface Modifications on Bioresorbable Bone Screws: A Step Forward in Orthopedic Surgery
Source: Polymers (Basel). 2025 Dec 24;18(1):52. doi: 10.3390/polym18010052 (PMC12788077; doi:10.3390/polym18010052)
Supplement: Supplementary file 1 [file polymers-18-00052-s001.zip › polymers-4029936-supplementary.pdf]

Supplementary material

Bioactive Surface Modifications on Bioresorbable Bone Screws: A Step Forward in Orthopedic Surgery

A. **G. Matveyeva, O. P. Boychenko, A. P. Moskalets, S. S. Zakakuev, N. A. Barinov, A. S. Bogdanova**[*]**, O. V. Morozova, D. V. Klinov**[*]**, D. A. Ivanov**

[*] Correspondence: as.bogdanova.as@gmail.com (A.S.B.), klinov.dv@mipt.ru (D.V.K.)

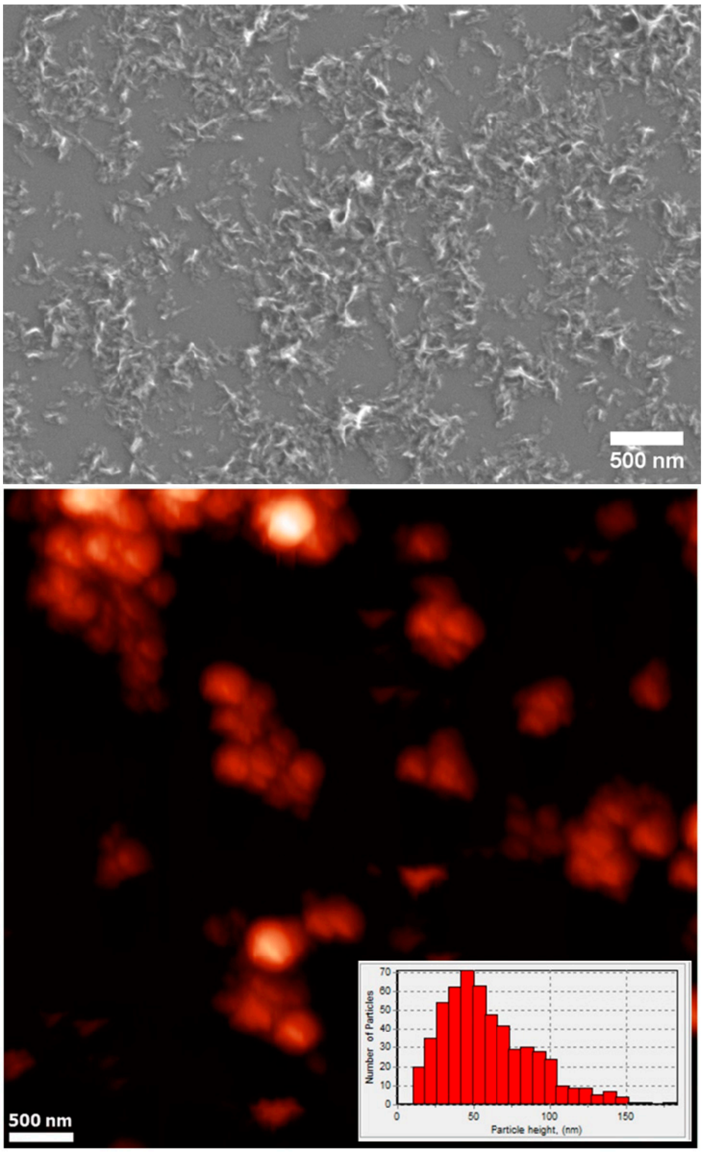

Figure S1. SEM image of hydroxyapatite nanoparticles (top) and AFM image of hydroxyapatite nanoparticles after the drying and redispersion procedure (bottom). The inset shows the height distribution of nHA obtained using AFM with a maximum at 50 ± 5 nm.

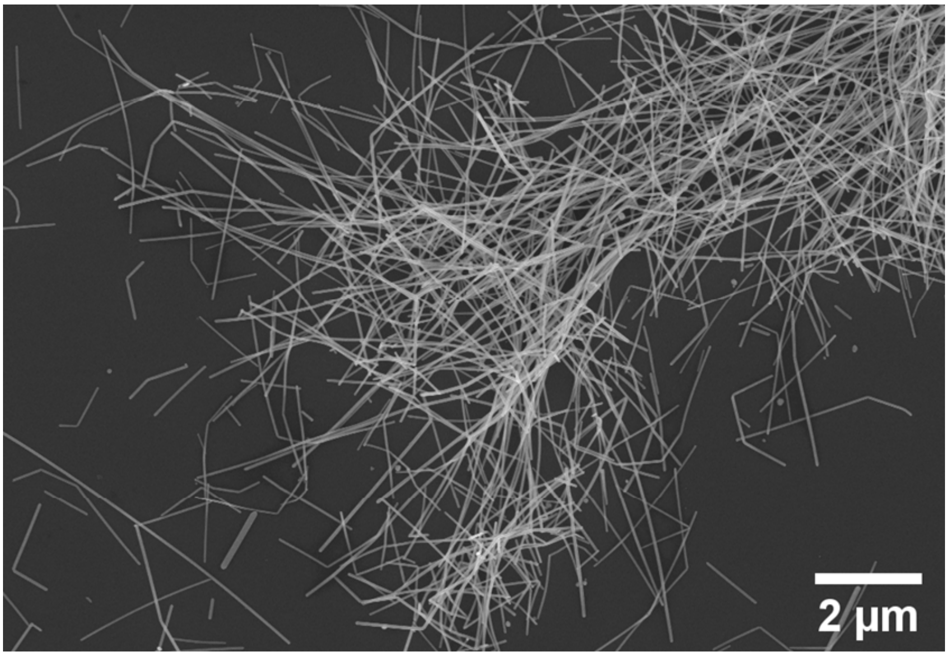

Figure S2. SEM image of silver nanowires

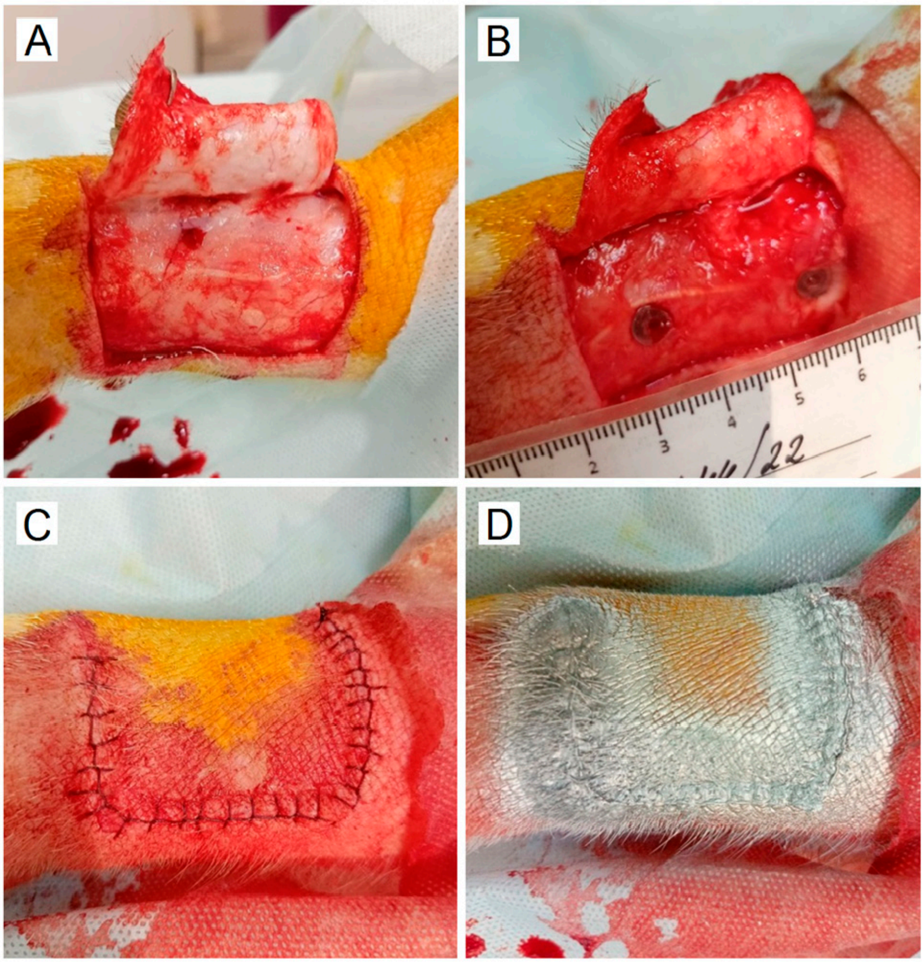

Figure S3. A) U-shaped incision, exposed surface of the metatarsal bone; B) Location of the implanted screws in the metatarsal bone; C) Closing the wound, suturing; D) Treatment of the seam and tissues adjacent to the seam with “Aluminum-Spray”.

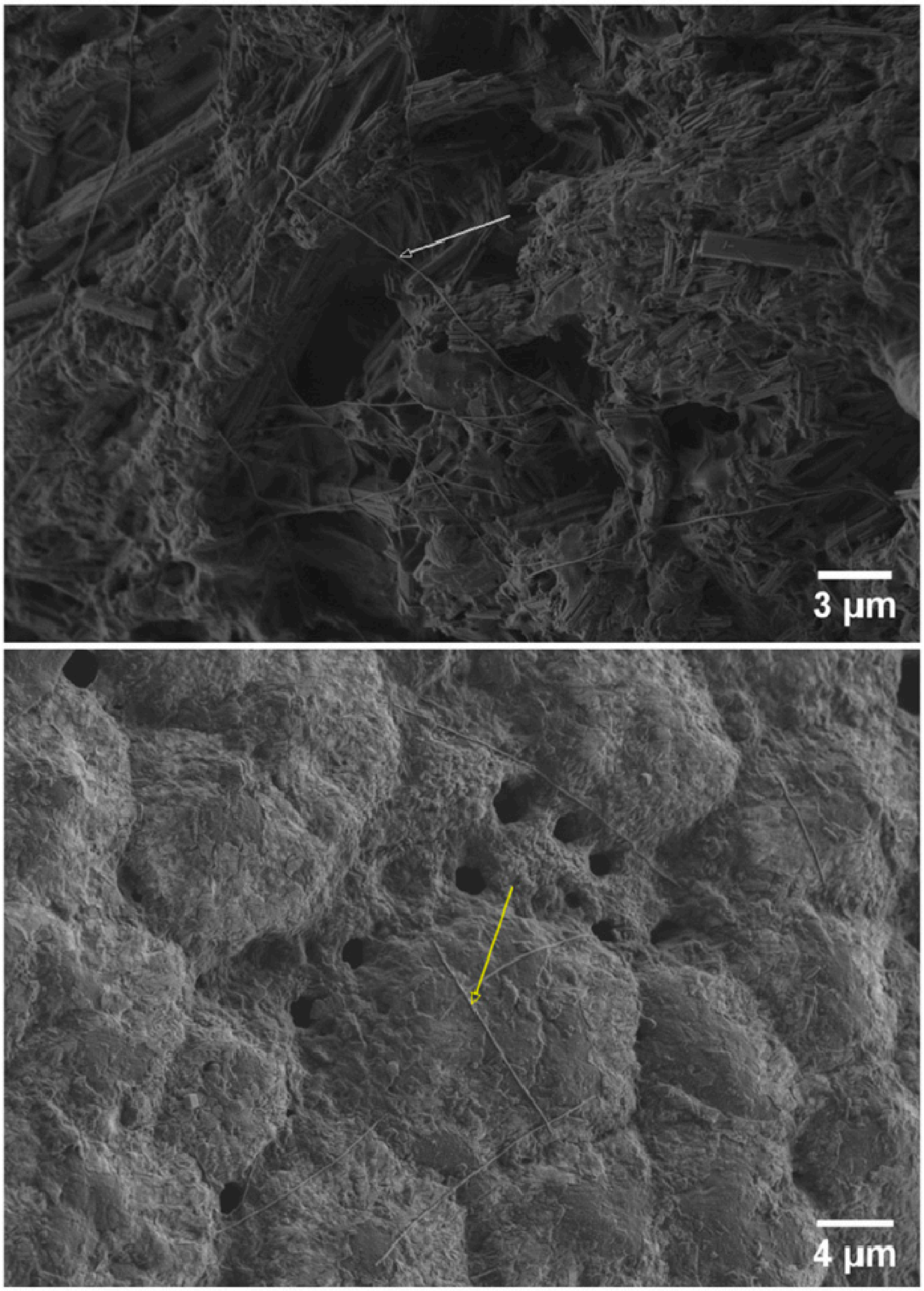

Figure S4. SEM image of the surface of thick (top) and thin (bottom) bioactive coatings with deposited silver nanowires. The positions of some nanowires are indicated by arrows.
